# The prevalence of subclinical ADHD and its associations with mental health and academic attitudes

**DOI:** 10.1192/j.eurpsy.2023.796

**Published:** 2023-07-19

**Authors:** B. Gács, I. Greges

**Affiliations:** 1 Institute of Behavioural Sciences; 2 Department of Psychiatry and Psychotherapy, Univerity of Pécs, Medical School, Pécs, Hungary

## Abstract

**Introduction:**

ADHD has been studied less extensively in adults than in children over the years, even though the indications of it clearly affect academic attitudes and closely linked to depression and substance abuse.

**Objectives:**

Exploratory cross-sectional research was conducted to examine the prevalence of subclinical ADHD is among medical students and its correlations with substance abuse. Furthermore, our goal was to find psychological and academic mediating variables, that might be risk or protective factors of its occurrence.

**Methods:**

A total of 239 (69 male) medical students were screened by an online questionnaire using Adult Self-Report Scale (ASRS), Depression, Anxiety and Stress Scale-21 (DASS-21), Maslach Burnout Inventory-Student Version (MBI-SS), Utrecht Work Engagement Scale for Students (UWES-S) and CAGE Questionnaire, which included smoking, alcohol, stimulant and sedative use.

**Results:**

Problematic substance use was reported by 48% of medical students for alcohol use, 43% for smoking, 25% for stimulant use and 21% for sedative use. The prevalence of ADHD symptoms is relatively high among medical students (m=36.13). Correlation and linear regression analysis showed a strong association between ADHD symptoms, depression, and substance abuse. The prevalence of subclinical ADHD symptoms mediates the relationship between depression and substance use, such as alcohol and stimulant use, but there is no significant relationship between academic attitudes (engagement and burnout) and ADHD symptoms, except for depersonalization, which was found to be a risk factor for the development of alcohol and stimulant use.

**Conclusions:**

The increased exposure of medical students to stress makes it particularly important to identify and address factors that can lead to more serious mental illness.

**Disclosure of Interest:**

None Declared

